# *Cs*severin inhibits apoptosis through mitochondria-mediated pathways triggered by Ca^2 +^ dyshomeostasis in hepatocarcinoma PLC cells

**DOI:** 10.1371/journal.pntd.0006074

**Published:** 2017-11-10

**Authors:** Mengchen Shi, Lina Zhou, Lu Zhao, Mei Shang, Tongtong He, Zeli Tang, Hengchang Sun, Pengli Ren, Zhipeng Lin, Tingjin Chen, Jinyun Yu, Jin Xu, Xinbing Yu, Yan Huang

**Affiliations:** 1 Department of Parasitology, Zhongshan School of Medicine, Sun Yat-Sen University, Guangzhou, China; 2 Key Laboratory for Tropical Disease Control, Ministry of Education, Sun Yat-Sen University, Guangzhou, China; 3 Guangdong Provincial Key Laboratory of Liver Disease Research, The Third Affiliated Hospital of Sun Yat-sen University, Guangzhou, China; 4 Guangdong Provincial Engineering Technology Research Center for Biological Vector Control, Guangzhou, China; 5 School of Public Health, Sun Yat-Sen University, Guangzhou, China; Istituto Superiore di Sanità, UNITED STATES

## Abstract

**Background:**

Numerous experimental and epidemiological studies have demonstrated a link between *Clonorchis sinensis* (*C*. *sinensis*) infestation and cholangiocarcinoma (CCA) as well as hepatocellular carcinoma (HCC). The underlying molecular mechanism involved in the malignancy of CCA and HCC has not yet been addressed. *Cs*severin, a component of the excretory/secretory products of C. *sinensis* (*Cs*ESPs), was confirmed to cause obvious apoptotic inhibition in the human HCC cell line PLC. However, the antiapoptotic mechanism is unclear. In the present study, we investigated the cellular features of the antiapoptotic mechanism upon transfection of the *Cs*severin gene.

**Methods:**

In the present study, we evaluated the effects of *Cs*severin gene overexpression on the apoptosis of PLC cells using an Annexin PE/7-AAD assay. Western blotting was applied to quantify the activation of caspase-3 and caspase-9, the mitochondrial translocation of Bax and the release of Cyt c upon *Cs*severin overexpression in PLC cells. Laser scanning confocal microscopy was used to analyze the changes of intracellular calcium. Fluorescence assay and immunofluorescence assays were performed to observe the changes of the mitochondrial permeability transition pore (MPTP).

**Results:**

The overexpression of *Cs*severin in PLC cells showed apoptosis resistance after the induction of apoptosis. Additionally, the activation of caspase-3 and caspase-9 was specifically weakened in *Cs*severin overexpression PLC cells. The overexpression of *Cs*severin reduced the increase in intracellular free Ca2+, thereby inhibiting MPTP opening in PLC cells. Moreover, Bax mitochondrial translocation and the subsequent release of Cyt c were downregulated in apoptotic *Cs*severin overexpression PLC cells.

**Conclusions:**

The present findings suggest that *Cs*severin, a component of *Cs*ESPs, confers protection from human HCC cell apoptosis via the inactivation of membranous Ca^2+^ channels. *Cs*severin might be involved in the process of HCC through *C*. *sinensis* infestation in affected patients.

## Author summary

Multiple studies have contributed to the association between *Clonorchis sinensis* (*C*. *sinensis*) infestation and cholangiocarcinoma (CCA) as well as hepatocellular carcinoma (HCC) in past years. However, studies on the underlying pathogenic mechanisms of *C*. *sinensis* lag behind those of other parasitic diseases. The excretory/secretory products of *C*. *sinensis* (*Cs*ESPs) are pathogenic, as these products promote cell proliferation, suppress cell apoptosis and stimulate inflammation. *Cs*severin, a component of *Cs*ESPs, inhibited the apoptosis of the human HCC cell line PLC in our previous study. The present study illustrated that *Cs*severin conferred human HCC cells protection from apoptosis via an intrinsic pathway (mitochondrial-mediated) triggered by the inactivation of membranous Ca^2+^ channels.

## Introduction

*Clonorchis sinensis* (*C*. *sinensis*) causes clonorchiasis, which is widely distributed in East Asia with heavily endemic zones in China, Taiwan, Vietnam, Russia, and Korea[1]. *C*. *sinensis* was reclassified as a group-I biocarcinogen for cholangiocarcinoma (CCA) by the International Agency for Research on Cancer (IARC) in 2009[2]. In endemic areas of China, 16.44% of hepatocellular carcinoma (HCC) patients were infected with *C*. *sinensis*, while 2.40% of non-tumor patients were infected [3]. This biocarcinogen has been included in control programs of neglected tropical diseases by the WHO[4]. The illumination of the precise mechanism linking *C*. *sinensis* with the development of HCC and CCA will help to prevent or postpone disease progression. Excretory-secretory proteins from *C*. *sinensis* (CsESPs) play important roles in the interactions between the worm and host, including the pathogenesis of inflammation, immune responses and carcinogenesis induced by the infection.

Escaping from apoptosis is an important aspect of cancer pathogenesis and has been widely recognized as a trait of most types of cancer [5]. In a previous study, we observed that *Cs*severin (a component of *Cs*ESPs), a homologous protein of the gelsolin family, caused obvious apoptotic inhibition in the human HCC cell line PLC. By promoting apoptosis suppression, *Cs*severin might accelerate the progress of HCC patients combined with *C*. *sinensis* infection [6]. It is worth studying the exact molecular mechanisms involved in the anti-apoptotic effects induced by *Cs*severin.

The gelsolin family had been implicated in the regulation of cell motility, apoptosis and phagocytosis [7]. The expression of gelsolin family proteins is reduced in many cancers, associated with poor prognosis and therapy resistance [8–9]. There is now increasing evidence that gelsolin family proteins are multifunctional regulators of cell apoptosis and cell metabolism, which involves multiple mechanisms[10–14].

Apoptosis can be executed in two distinct signaling cascades: the extrinsic pathway and the intrinsic pathway[15–16]. In the extrinsic pathway, apoptosis is triggered by death receptors, such as FAS-associated death domain protein (FADD), activating caspases 8 and 10 (the initiator caspases), which in turn activate executioner caspases 3, 6 and 7[17]. In the intrinsic pathway, the mitochondrial permeability transition pore (MPTP) plays a pivotal role in regulating the release of pro-apoptotic proteins, such as cytochrome c (Cytc). The released Cytc from mitochondria initiated the assembly of apoptosomes, activating factor 1 (Apaf-1) and caspase 9, an initiator caspase that cleaves and activates caspase 3 and 7[18]. MPTP is regulated by Bcl-2 proteins that induce the oligomerization of BAX (Bcl-2-associated protein) or BAK (Bcl-2 antagonist)[19].

The results of a previous study indicated that *Cs*severin binds to calcium ions in solution and actin filaments inside cells. We also demonstrated that the co-incubation of PLC cells with *Cs*severin *in vitro* led to apoptosis suppression based on the detection of the apoptosis-associated changes of mitochondrial membrane potential[6]. To further understand the anti-apoptotic role of *Cs*severin, we constructed stable *Cs*severin-overexpressing PLC cells (pEZ-LV203-*Cs*severin PLC) to avoid interference from endotoxin through the use of recombinant *Cs*severin. We detected a suppression effect of *Cs*severin on the early wave apoptosis of PLC cells. Furthermore, to investigate the mechanisms involved in *Cs*severin induced apoptosis suppression, we explored the effects of *Cs*severin on the activation of the caspase cascade, leading to the suppression of the permeability transition pore (MPTP), the mobilization of calcium, and the translocation of Cyt c and Bax.

## Methods

### Ethics statement

The Ethics Committee of Sun Yat-Sen University reviewed and approved the protocols and experiments used in this study. The methods were carried out in accordance with the approved protocols. The data were collected and analyzed anonymously.

### Cell culture

The human HCC cell line PLC were a gift from Dr. Wang Shutong (the First Affiliated Hospital of Sun Yat-Sen University) and routinely cultured in high glucose DMEM medium (Gibco, USA) supplemented with 10% fetal bovine serum (Gibco, USA) and penicillin-streptomycin (100 units/ml) in 5% CO_2_ at 37°C. The human 293T cells were kindly provided by GeneCopoeia (Rockville, MD, USA) and maintained in high glucose DMEM supplemented with 10% fetal bovine serum in 5% CO_2_ at 37°C.

### Antibodies

Cox-IV, Caspase 3, Caspase 9, Bax and Cytochrome c were purchased from Cell Signaling Technology (Danvers, MA, USA). β-actin was obtained from Proteintech (USA). Anti-*Cs*severin serum was prepared as previously described [6].

### Construction and identification of PLC cells stably overexpressing *Cs*severin

The pEZ-LV203 lentiviral vector harboring the eGFP reporter gene was purchased from GeneCopoeia (Rockville, MD, USA). The pEZ-LV203 vector and *Cs*severin gene fragments were digested with *EcoR*I and *Apa* I, respectively, and subsequently ligated using T4 DNA ligase. The recombinant plasmid pEZ-LV203-*Cs*severin was identified by enzyme digestion and sequencing.

To generate the lentivirus, the pEZ-LV203-*Cs*severin plasmid or PEZ-LV203 control plasmid was cotransfected into 293T cells using the Lenti-Pac HIV Expression Packaging Kit (GeneCopoeia, USA) according to the manufacturer’s instructions. Supernatant containing the recombinant lentiviral particles was collected at 48 h post-transfection, filtered by a Millipore filter and subjected to ultracentrifugation. The lentiviral particles were re-suspended in cold phosphate-buffered saline (PBS) and used to infect PLC cells. The PLC cells were divided into three groups, pEZ-LV203-*Cs*severin PLC (transfected with pEZ-LV203-*Cs*severin plasmid), pEZ-LV203 PLC (PEZ-LV203 control plasmid) and PLC (no transfection). After 48 h, the cells were incubated in selection medium containing puromycin (3 mg/ml) for 7 days to select stably *Cs*severin-overexpressing PLC cells (pEZ-LV203-*Cs*severin PLC) and control PLC cells (pEZ-LV203 PLC). The transfection efficiency of pEZ-LV203-*Cs*severin PLC was evaluated by the expression of eGFP, and the *Cs*severin protein expression levels of pEZ-LV203-*Cs*severin PLC were measured by Western blot analysis.

http://dx.doi.org/10.17504/protocols.io.kcdcss6[PROTOCOL DOI]

### Analysis of apoptosis by AnnexinPE/7-aminoactinomycin D (7-AAD) staining

Apoptotic cells were assessed by Annexin PE/7-AAD detection as previously described [6]. Briefly, Control groups (pEZ-LV203 PLC and PLC) or pEZ-LV203-*Cs*severin PLC cells were plated at a density of 10^5^ cells per well in 6-well plates, and apoptosis was spontaneously induced after serum starvation for 48 h. The cells were collected by centrifugation, washed with cold PBS, and subsequently resuspended in 500 μl of 1× Binding Buffer prior to incubation with 5 μl of Annexin PE and 5 μl of 7-AAD (Keygentec, Nanjing, China). The cell samples were incubated at room temperature for 20 min and subsequently detected by a flow cytometer (Beckman Coulter Gallios, USA) to determine the apoptotic cell fractions.

http://dx.doi.org/10.17504/protocols.io.kcecste[PROTOCOL DOI]

### Preparation of total protein extraction

The pEZ-LV203-*Cs*severin PLC cells were pretreated by serum starvation for 48 h, and pEZ-LV203 PLC and PLC cells were used as controls. A total of 5 × 10^6^ cells were collected and treated with 300 μl of RIPA buffer (150 mM NaCl, 50 mM Tris, pH 7.4, 1% NP40, 0.1% SDS, and 0.5% sodium deoxycholate) supplemented with protease and phosphatase inhibitors (Keygentec, Nanjing, China).

### Isolation of cell fractionation

To monitor the shift in Cytc from the mitochondria and Bax from the cytosol, we fractionated the cytosolic and mitochondrial fractions using a Cell Mitochondria Isolation Kit according to the manufacturer's instructions (Beyotime Institute of Biotechnology, China). The pEZ-LV203-*Cs*severin PLC cells were pretreated by serum starvation for 48 h, and pEZ-LV203 PLC and PLC cells were used as controls. A total of 5 × 10^6^ cells were collected after brief trypsinization, followed by two more washes with PBS, and the cell pellet was resuspended in 200 μl of mitochondria extraction buffer containing 0.02 mM phenylmethanesulfonyl fluoride (PMSF) and proteinase inhibitors (Keygentec, Nanjing, China). After incubating on ice for 20 min, the cells were homogenized using a glass Dounce and pestle. The homogenates were centrifuged at 600 g for 15 min at 4°C, and the resulting supernatant was collected and centrifuged at 11,000 g for 15 min at 4°C to separate the mitochondria (pellet) and cytoplasmic proteins (supernatant). The mitochondria pellet was lysed in mitochondria extraction buffer (KeyGen Biotech, Nanjing, China).

http://dx.doi.org/10.17504/protocols.io.kcecstedx.doi.org/10.17504/protocols.io.kcfcstn [PROTOCOL DOI]

### Analysis of protein levels by western blotting

Western blotting analysis to determine the levels of apoptosis-related proteins was performed using standard techniques. The concentration of protein was determined by the BCA protein assay kit (Beyotime Institute of Biotechnology, China). Equal amounts of protein were subjected to Western blotting analysis. The proteins (40 μg) were separated according to molecular weight on a 12% SDS-PAGE gel and transferred onto a polyvinylidene difluoride (PVDF) membrane. The membranes were blocked with 1% bovine serum albumin in Tris-Buffered Saline Tween-20 (TBST, pH 7.4) at room temperature for 2 h, and probed overnight at 4°C with specific primary antibodies at the following dilutions: β-actin and Cox-IV, 1:2000; anti-*Cs*severin sera, 1:100; caspase 3 and caspase 9, 1:1000; and Bax and Cyt c, 1:500. After washing with TBST, the membranes were incubated with goat-anti-mouse or goat-anti-rabbit horseradish peroxidase-conjugated secondary antibody (1:5000) for 1 h at room temperature. Immunoreactive bands were visualized by the enhanced chemiluminescence detection kit (KeyGen Biotech, Nanjing, China) and quantified using the Gel-pro 4.5 Analyzer (Media Cybernetics, USA).

### Measurement of intracellular Ca^2+^

The intracellular Ca^2+^ concentration was estimated by co-incubating the cells with a cell-permeant Ca^2 +^ fluorophore, Rhod-2 AM (2 μM). PEZ-LV203-*Cs*severin PLC cells were seeded at a density of 10^2^ cells onto a confocal culture dish and treated by serum starvation for 48 h, and pEZ-LV203 PLC and PLC cells were used as controls. The cells were washed with cold PBS and incubated in a 5% CO_2_ humidified incubator at 37°C for 20 min after adding 20 μl of Rhod-2 AM working solution (AAT Bioquest, USA). Next, the cells were washed twice with PBS and the changes of intracellular calcium were evaluated by a laser scanning confocal microscope (Zeiss LSM 710, Germany). The Rhod-2 AM fluorescence was observed at 525 nm excitation (Ex)/590 nm emission (Em).

http://dx.doi.org/10.17504/protocols.io.kcecstedx.doi.org/10.17504/protocols.io.kcgcstw[PROTOCOL DOI]

### Detection of mitochondrial permeability transition pore (MPTP)

The mitochondrial permeability transition pore (MPTP) was detected by tetramethyl rhodamine methyl ester (TMRM) in the Cell MPTP assay kit (Genmed Scientific Inc., Arlington, TX, USA). TMRM is a membrane-permeable fluorophore. In live cells, the hydrolysis of TMRM by intracellular esterases produces strongly red fluorescent tetramethyl rhodamine, a lipophilic compound well retained in cell mitochondria. The cytoplasm was stained with the methyl ester derivative of TMRM quenching of mitochondria rhodamine fluorescence. This nature of TMRM enables the assessment of MPTP opening[20].

Briefly, the control groups (pEZ-LV203 PLC and PLC) or pEZ-LV203-*Cs*severin PLC cells were seeded at a density of 10^3^ cells per well onto 6-well plates, and spontaneous apoptosis was induced through serum starvation for 48 h. The cells were rinsed with GENMED cleaning solution and incubated with 1 ml GENMED staining solution for 20 min at 37°C in the dark. The supernatant was subsequently discarded, and the cells were washed twice with GENMED cleaning solution. Subsequently, the changes of MPTP were monitored using an inverted fluorescence microscope (Leica DMI4000B, Germany).

Quantitative changes of MPTP during cell apoptosis were measured by flow cytometry with the TMRM probe. After induced spontaneous apoptosis by serum starvation for 48 h, 10^5^ cells were harvested and resuspended with GENMED cleaning solution. Subsequently, the cell suspensions were incubated with 0.5 ml of TMRM working solution for 20 min at 37°C in the dark. The staining solution was removed by centrifugation. The cells were washed twice with GENMED cleaning solution, subsequently resuspended in 200 μl of buffer solution and detected using a flow cytometer (Beckman Coulter Gallios, USA).

http://dx.doi.org/10.17504/protocols.io.kcecstedx.doi.org/10.17504/protocols.io.kchcst6[PROTOCOL DOI]

### Statistical analysis

The data were analyzed for statistical significance using SPSS 13.0 software (SPSS, Chicago, IL, USA). The results are expressed as the means±SD from at least 3 independent experiments performed in duplicate. Statistical comparisons of the results were performed using one-way analysis of variance (ANOVA). A *P* value < 0.05 was considered statistically significant.

## Results

### PLC cells stably overexpressing *Cs*severin

**Fig 1:**
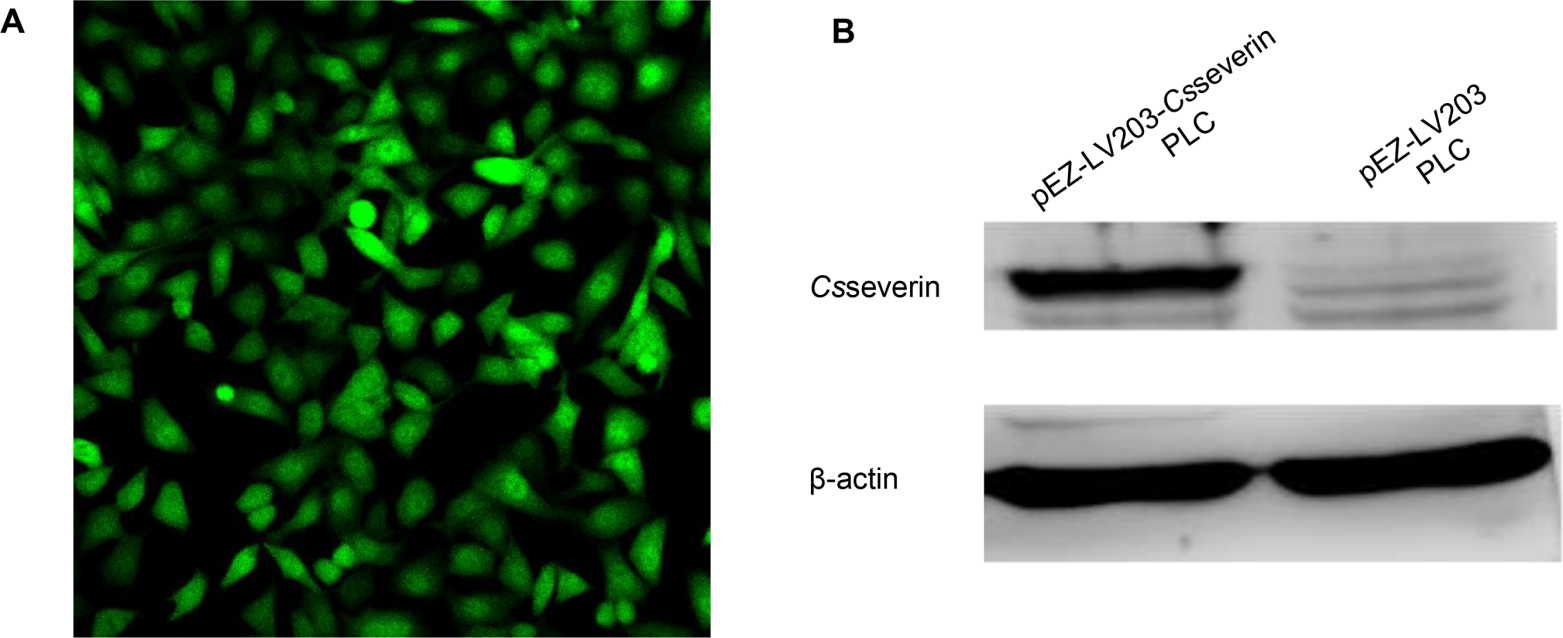
Identification of PLC cells stably overexpressing *Cs*severin. (A) The green fluorescence protein (GFP) in *Cs*severin overexpression PLC cells (pEZ-LV203-*Cs*severin PLC) by fluorescent microscopy under a 20X objective. (B) Western blotting analysis. Total proteins from pEZ-LV203-*Cs*severin PLC and control cells (pEZ-LV203 PLC) were subjected to SDS-PAGE and subsequently analyzed. Rat anti-r*Cs*severin serum was used as the primary antibody at a dilution of 1:100. The pEZ-LV203-*Cs*severin PLC cells were probed by rat anti-r*Cs*severin serum, detecting a band at approximately 45 kDa, while no corresponding band was observed in pEZ-LV203 PLC cells. β-actin was loaded as a control.

As shown in Fig 1A, green fluorescence was observed in pEZ-LV203-*Cs*severin PLC cells containing the pEZ-LV203-*Cs*severin vector fused with the eGFP reporter gene (Fig 1A). Compared to control PLC cells (pEZ-LV203 PLC), *Cs*severin expression was significantly increased in pEZ-LV203-*Cs*severin PLC cells, as shown by Western blotting (Fig 1B).

### Apoptosis suppression of pEZ-LV203-*Cs*severin PLC cells

**Fig 2:**
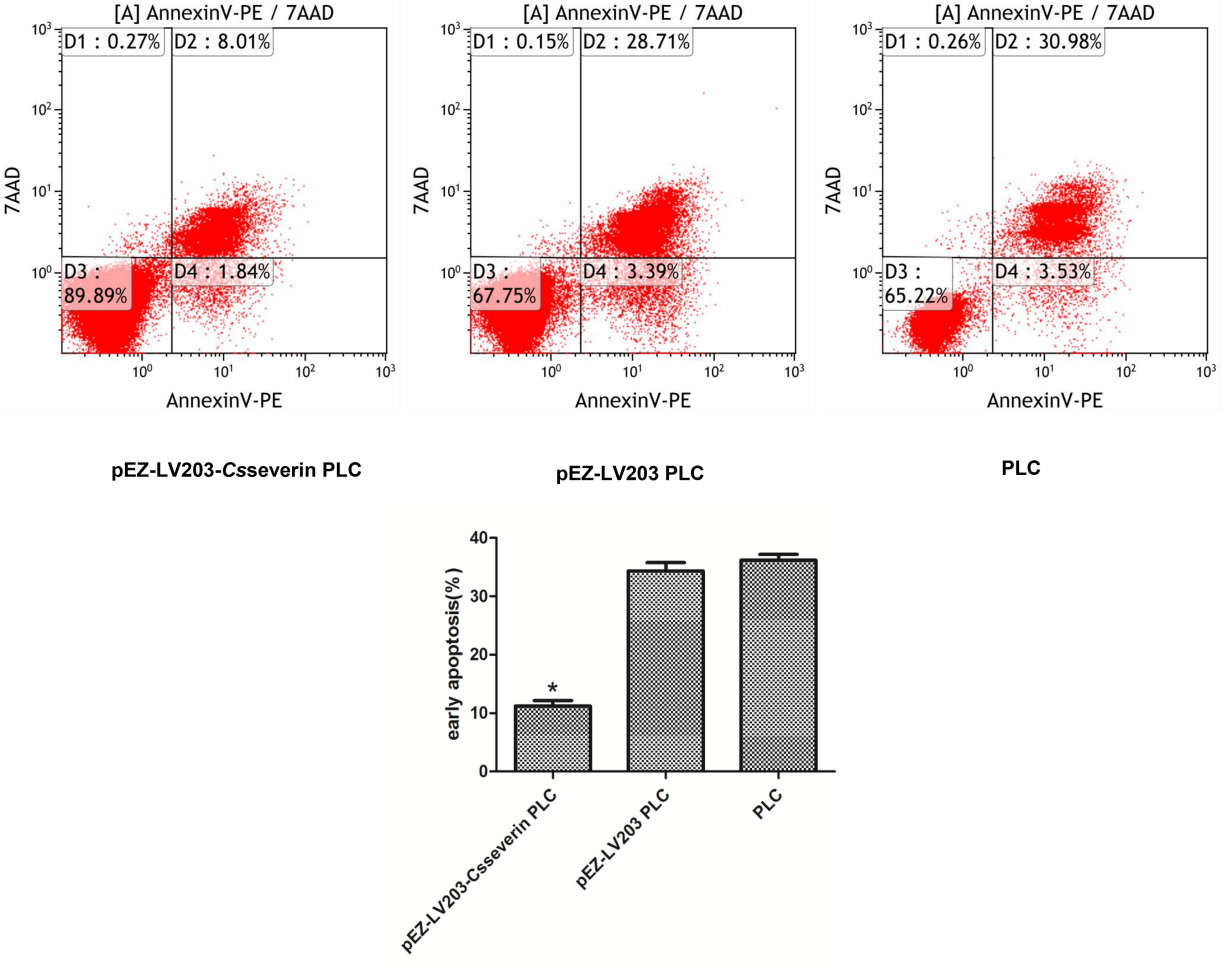
Overexpression of *Cs*severin inhibits the apoptosis of PLC cells. Apoptosis was analyzed by flow cytometry using an Annexin PE/7-AAD staining kit. After spontaneous apoptosis was induced by serum starvation for 48 h, pEZ-LV203-*Cs*severin PLC cells were stained with Annexin PE and 7-AAD and subsequently quantified by flow cytometry analysis. pEZ-LV203 PLC and PLC cells were used as controls.

We conducted an Annexin PE/7-AAD binding assay using flow cytometry and detected the total ratio of Annexin PE+/7-AAD- and Annexin PE+/7-AAD+ cells. The apoptotic ratio of pEZ-LV203-*Cs*severin PLC cells was 9.85%, obviously lower than that of the control cells (pEZ-LV203 PLC and PLC), which showed 32.1% and 34.51%, respectively (Fig 2).

### Expression levels of caspase 9 and caspase 3 in pEZ-LV203-*Cs*severin PLC cells

**Fig 3:**
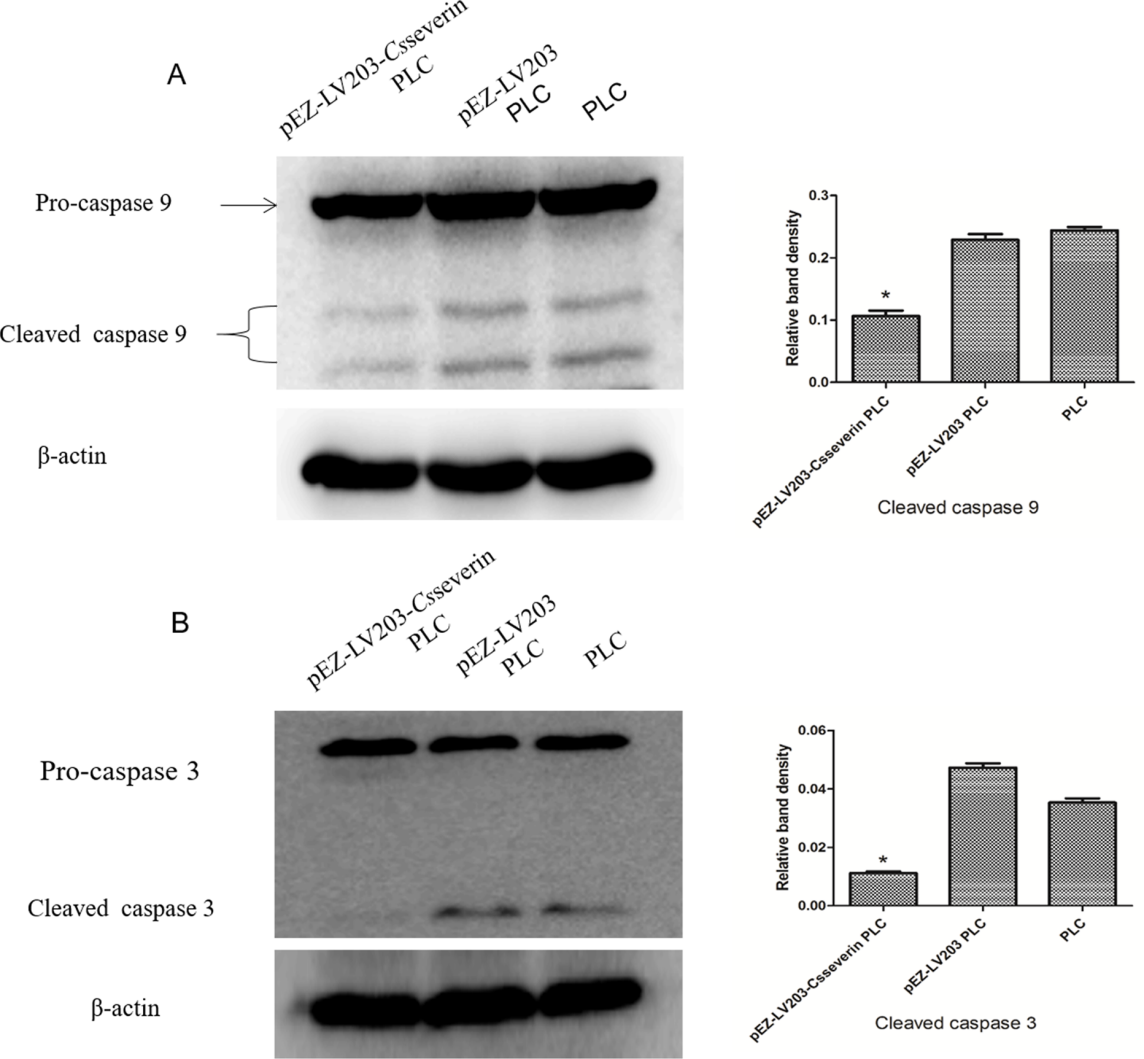
Overexpression of *Cs*severin inhibits caspase-dependent apoptosis in PLC cells. pEZ-LV203-*Cs*severin PLC cells were pretreated by serum starvation for 48 h, and pEZ-LV203 PLC and PLC cells were used as control groups. (A, B) The expression levels of caspase 3 and caspase 9 were examined by Western blotting, with β-actin loaded as a control. In *Cs*severin overexpression PLC cells (pEZ-LV203-*Cs*severin PLC), cleaved caspase 3 and cleaved caspase 9 expression was downregulated compared to that of control cells (pEZ-LV203 PLC and PLC). *P < 0.05, compared to control groups.

To determine the apoptotic pathways involved in the *Cs*severin-suppressed early wave of apoptosis, we further explored changes in the activities of initiator caspase (caspase 9) and effector caspase (caspase 3) by Western blot analysis. The results showed the accumulation of cleaved caspase 9 and cleaved caspase 3 in the control groups (pEZ-LV203 PLC and PLC), while expression levels of cleaved caspase 9 and cleaved caspase 3 were decreased in pEZ-LV203-*Cs*severin PLC cells (Fig 3, *P* < 0.05).

### Opening of the mitochondrial permeability transition pore (MPTP) in pEZ-LV203-*Cs*severin PLC cells

**Fig 4:**
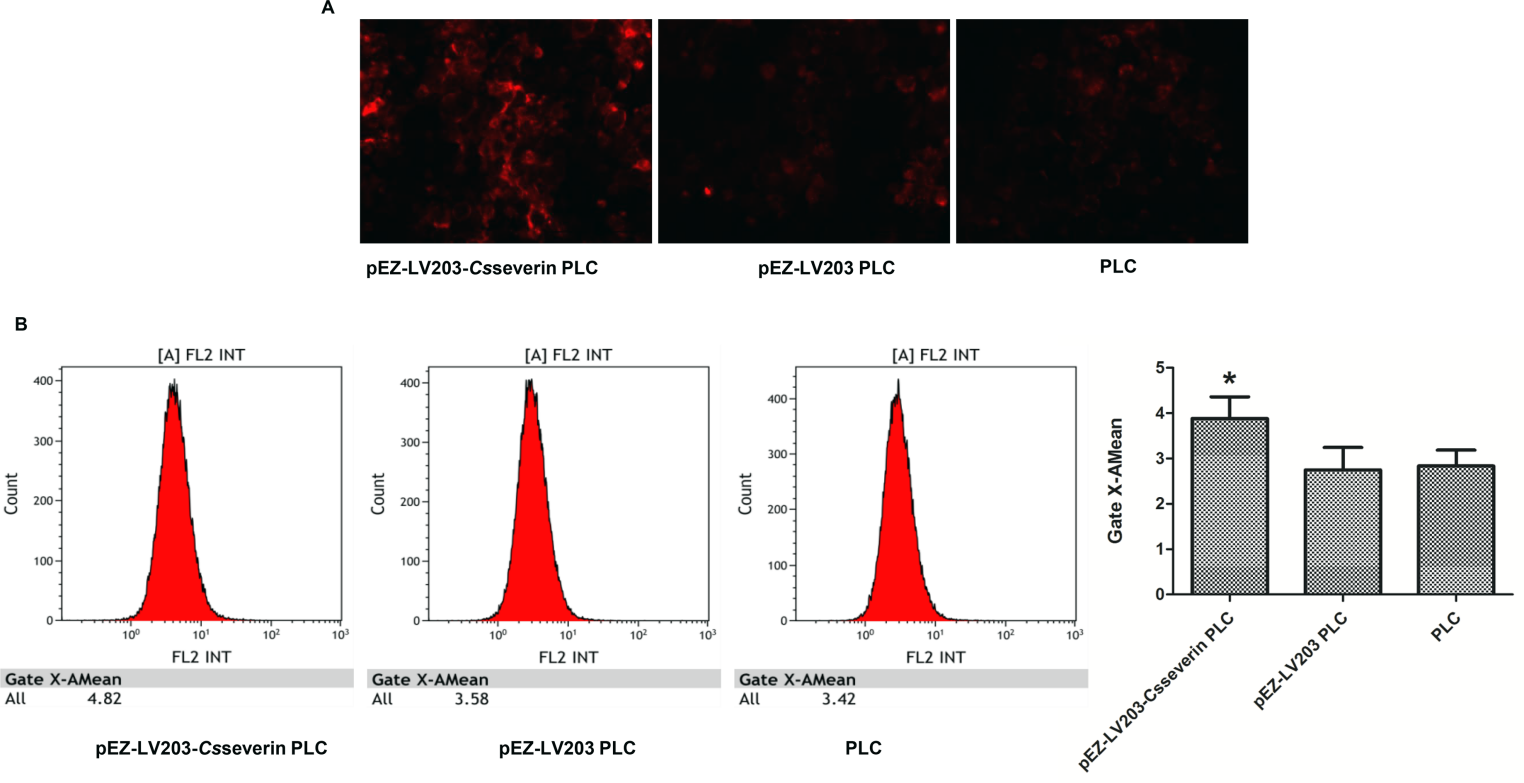
Effect of *Cs*severin on the mitochondrial permeability transition pore (MPTP) in PLC cells. After spontaneous apoptosis was induced by serum starvation for 48 h, Control groups (pEZ-LV203 PLC and PLC) or pEZ-LV203-*Cs*severin PLC cells were detected by tetramethylrhodamine methyl ester (TMRM). The suppression of MPTP opening was demonstrated by the enhanced red fluorescence in pEZ-LV203-*Cs*severin PLC cells. (A) Typical fluorescence photomicrograph of the TMRM staining output by a fluorescence microscope. (B) The quantitative analysis of MPTP was analyzed by flow cytometry upon staining with the fluorescent dye TMRM. *P < 0.05, compared to control groups.

The opening of the MPTP marks the irreversible point of cell apoptosis [21]; therefore, we examined whether the MPTP participates in the anti-apoptotic mechanism induced by *Cs*severin. TMRM revealed significantly enhanced red fluorescence intensity in *Cs*severin pEZ-LV203-*Cs*severin PLC cells compared with the control group (pEZ-LV203 PLC and PLC) (Fig 4A). The geometric mean, indicating the average red fluorescent intensity of pEZ-LV203-*Cs*severin PLC、pEZ-LV203 PLC or PLC cells emitting red fluorescence, was 4.82、3.58 and 3.42 (Fig 4B), respectively, suggesting decrease in MPTP opening.

### Effect of *Cs*severin on Ca^2+^ homeostasis

**Fig 5:**
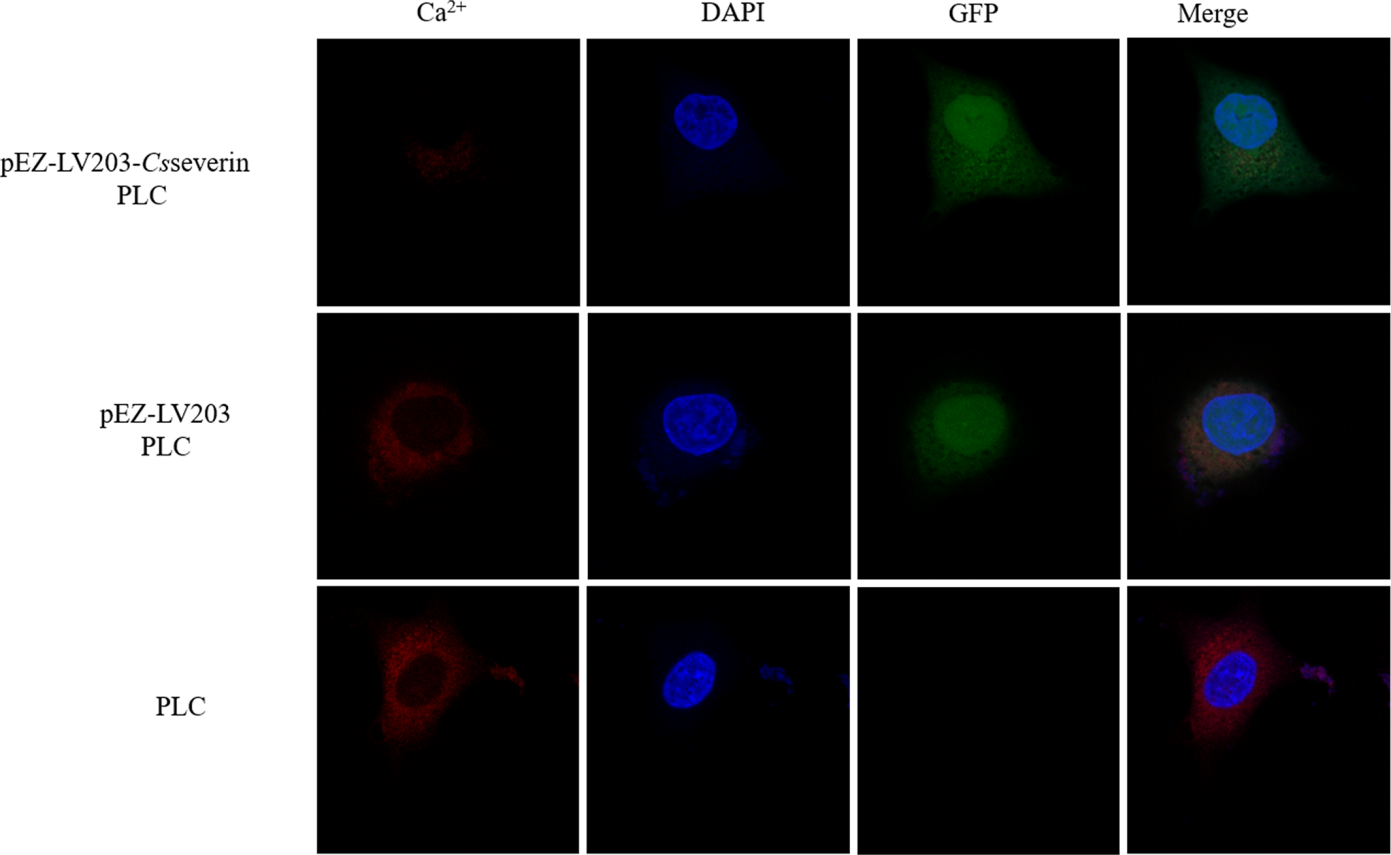
The intracellular distribution of free Ca^2+^ was analyzed by laser scanning confocal microscopy. Intracellular free Ca^2+^ was detected by red fluorescent probe dihydrorhod-2 AM (Rhod-2 AM). The nuclei were stained with DAPI (blue). The pEZ-LV203 vector harboring the eGFP reporter gene produced green fluorescent protein.

In a previous study, we showed that *Cs*severin binds to Ca^2+^ *in vitro*. Since Ca^2+^ has been demonstrated as a key substrate associated with apoptosis in different cell types [22], and MPTP has been recognized as a major target of Ca^2+^[21], we further confirmed whether *Cs*severin-inhibited apoptosis was associated with Ca^2+^ imbalance in PLC cells. The cells were stained with the fluorescent probe dihydrorhod-2 AM (Rhod-2 AM) for the analysis of intracellular free calcium. The concentration of intracellular free Ca^2+^ obviously increased in the control groups, pEZ-LV203 PLC and PLC, while intracellular free Ca^2+^ was predominantly reduced in pEZ-LV203-*Cs*severin PLC cells (Fig 5).

### Expression levels of Cyt c and Bax in the cytoplasm and mitochondria of pEZ-LV203-*Cs*severin PLC cells

**Fig 6:**
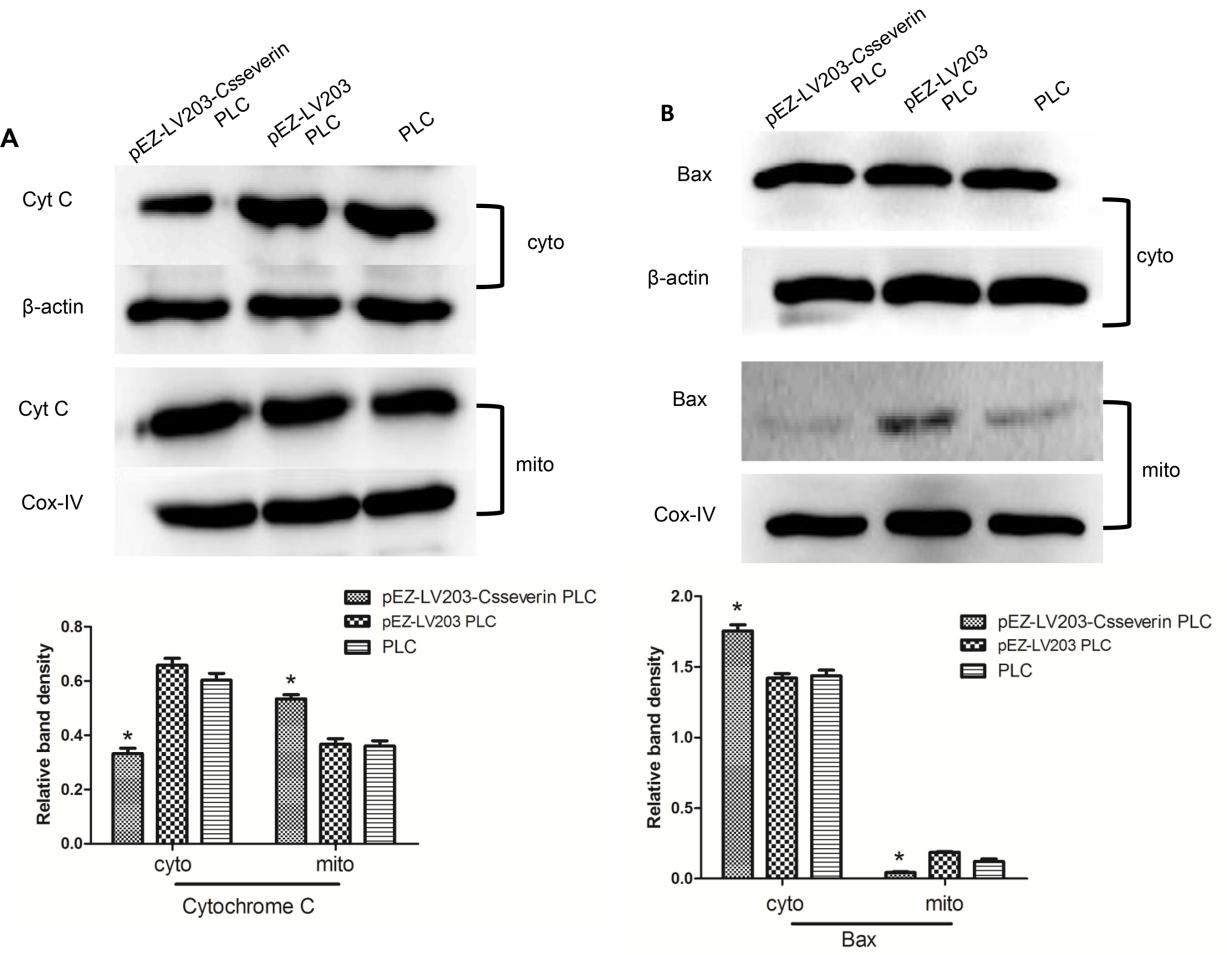
Effects of *Cs*severin on major apoptotic molecules in PLC cells. The spontaneous apoptosis of pEZ-LV203-*Cs*severin PLC cells and control groups (pEZ-LV203 PLC and PLC) was induced through serum starvation for 48 h. The cells were fractionated to obtain mitochondria and cytosol subfractions. β-actin was loaded as a control for the cytosol subfraction, and cox-IV was loaded as a control for the mitochondria subfraction. (A) The intracellular location of Cyt c was determined by Western blot analysis. Cyt c release from the mitochondria was inhibited in *Cs*severin overexpression PLC cells (pEZ-LV203-*Cs*severin PLC). (B) Western blotting showed that compared with control cells (pEZ-LV203 PLC and PLC), pEZ-LV203-*Cs*severin PLC showed a drastic reduction in the mitochondrial translocation of Bax. *P < 0.05, compared to control groups.

The loss of mitochondrial membrane potential induces mitochondrial permeability by opening the MPTP, which primarily initiates the translocation of the apoptogenic protein Cyt c from mitochondria into the cytoplasm[23]. Subcellular fractionation was performed to examine Cyt c levels in both cytosolic and mitochondrial compartments. Compared to those of the control groups, the significant downregulation of cytoplasmic Cyt c expression and the upregulation of mitochondrial Cyt c expression in pEZ-LV203-*Cs*severin PLC cells were observed, indicating an inhibitory effect on the release of Cyt c from mitochondria into the cytoplasm (Fig 6A, *P* < 0.05).

The mitochondrial translocation of Bax is a key step that prompts the release of Cyt c from the mitochondria[24]. Western blot analysis showed that compared with control cells (pEZ-LV203 PLC and PLC), *Cs*severin overexpression PLC cells (pEZ-LV203-*Cs*severin PLC) showed a drastic reduction in the translocation of Bax to mitochondria (Fig 6B, *P* < 0.05).

## Discussion

Previous studies have shown that *Cs*severin could induce apoptotic inhibition in spontaneously apoptotic human HCC PLC cells. In the present study, we confirmed the anti-apoptotic role of *Cs*severin and explored the involved mechanisms. We generated stably *Cs*severin-overexpressing PLC cells (PEZ-LV203-Cssevein PLC) and control cells (PEZ-LV203 PLC) in the present study. The results demonstrated significant suppression during the early period of apoptosis in pEZ-LV203-*Cs*severin PLC cells compared with pEZ-LV203-PLC and PLC cells.

Apoptosis occurs via two different pathways: the extrinsic pathway (death receptors) and the intrinsic pathway (mitochondria and endoplasmic reticulum)[15–16]. In a previous study, we observed that *Cs*severin led to the recovery of mitochondrial membrane potential (MMP) in PLC cells and speculated that the mitochondrial signal pathway may be involved in *Cs*severin-mediated protection from apoptosis.

Mitochondria are sensitive to the external environment, responding with MMP alterations that lead to the release of apoptosis-related factors and cell apoptosis[25]. There are several specific proteins in the mitochondrial-mediated pathway. Caspase 3 and caspase 9 are the key factors associated with the mitochondrial-mediated pathway. Caspase 9 activity is primarily dependent on the intrinsic pathway (mitochondrial-mediated) regulated by members of the Bcl-2 family[26]. In the present study, compared with control cells (PLC and pEZ-LV203 PLC), we observed a decrease in caspase 9 activity in spontaneously apoptotic pEZ-LV203-*Cs*severin PLC cells. The reduced activation of caspase 9 subsequently suppressed downstream caspase 3, which was activated through the mitochondrial-mediated pathway. Therefore, these results suggested that via intrinsic (mitochondrial-mediated), extrinsic (death receptors) or other intrinsic (endoplasmic reticulum) pathways, *Cs*severin might confer protection from the early wave of apoptosis in PLC cells.

Bax, a proapoptotic member of the Bcl-2 family proteins, is an initiator in the mitochondrial-mediated pathway[27]. In healthy living cells, Bax is predominantly located in the cytosol and migrates to the mitochondrial membrane during early apoptosis[28]. This translocation induced Cyt c release from mitochondria to the cytoplasm [29]. Cyt C can combine with procaspase 9 and Apaf-1 to form an apoptosome to activate caspase-9 and other caspases that induce the downstream caspase cascade. We detected the mitochondrial translocation of Bax and the release of Cyt c. The present study showed that the overexpression of *Cs*severin significantly suppressed the mitochondrial translocation of Bax, followed by the decreased release of Cyt c from mitochondria.

The gelsolin family (include *Cs*severin) plays a leading role in controlling actin filament reorganization/remodeling[12]. In several models of cell apoptosis, gelsolin has demonstrated an anti-apoptotic property associated with its effects on the dynamic actin cytoskeleton by preventing the loss of mitochondrial membrane potential and activation of caspase 3[30–31]. The organization/remodeling of actin filaments can also release Ca^2+^ from the F-actin store and open the influx pathway for the external release of Ca^2+^ into the cell[32]. Intracellular Ca^2+^ is used as a second messenger to regulate most crucial biological processes, such as cell survival, proliferation and gene transcription[33]. In some experimental systems, the elevation of intracellular Ca^2+^ levels is regarded as a pivotal element of apoptosis[34–35]. Thus, the Rhod-2 AM Ca^2+^ fluorophore, which emits red fluorescence, was used to evaluate changes of intracellular Ca^2+^. Previous studies have shown that *Cs*severin binds to Ca^2+^ and cytoskeletal actin filaments [6]. The results of the present study showed a significant decrease of intracellular calcium in *Cs*severin overexpression PLC cells, associated with the effect of *Cs*severin on apoptosis suppression.

The intracellular Ca^2+^ level is affected by mitochondrial Ca^2+^ sequestration, which might eventually stimulate the prolonged opening of the MPTP. MPTP is a multi-protein complex formed between mitochondrial membranes, and persistent MPTP opening results in the osmotic dysregulation of the mitochondrial membrane[36]. Once the MPTP is opened, various apoptosis-related proteins, such as Bax, could enter mitochondria and lead to a decrease of the mitochondrial membrane potential, the release of Cyt c, and the induction of early apoptosis[37]. We also measured the changes in MPTP using a TMRM probe. Compared with PLC and pEZ-LV203 PLC cells (negative control), enhanced fluorescence intensity was observed in pEZ-LV203-*Cs*severin PLC cells after induced spontaneous apoptosis by serum-starvation for 48 h, indicating the inhibition of MPTP opening.

**Fig 7:**
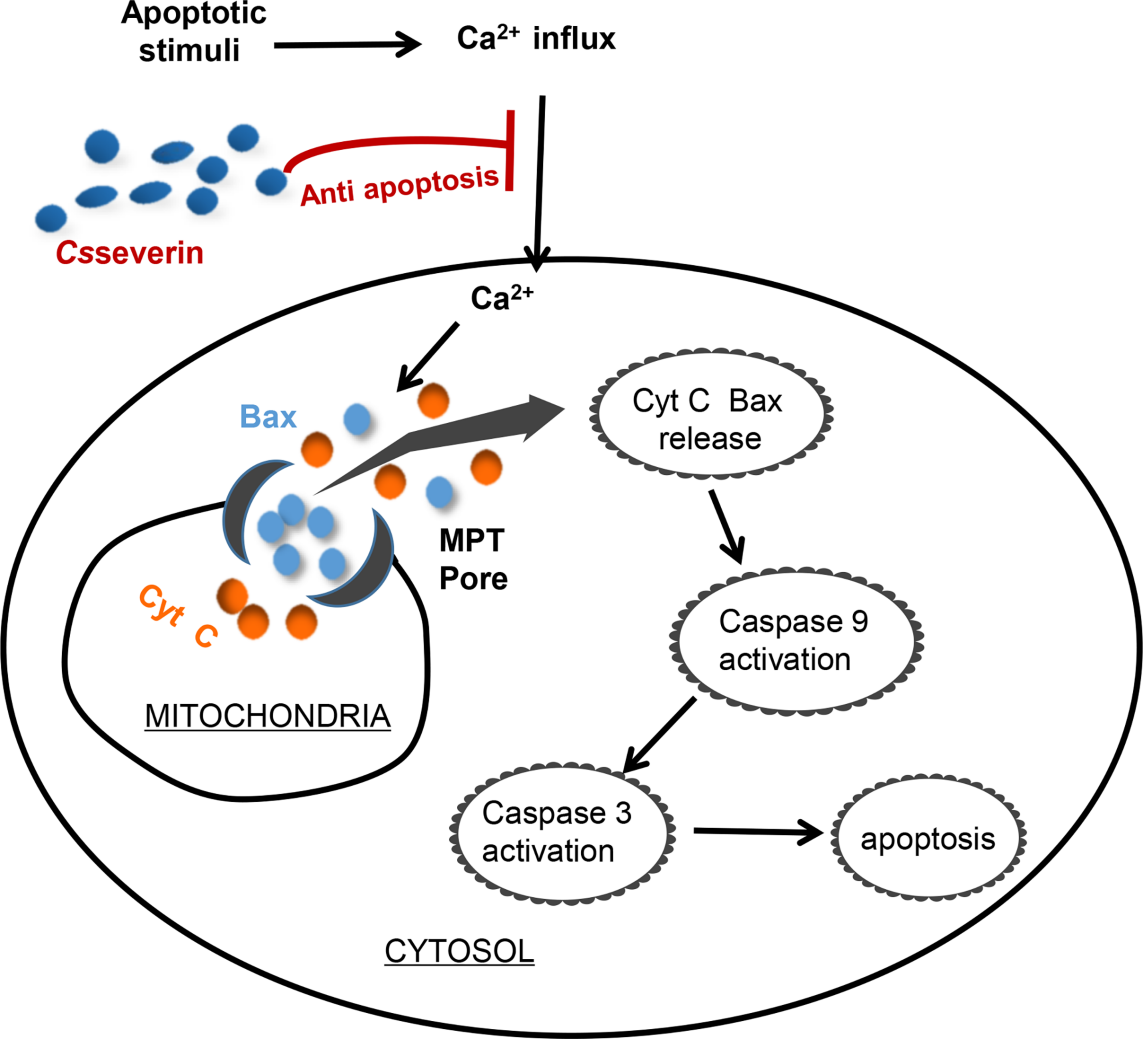
Model of the potential anti-apoptotic mechanism induced by *Cs*severin. *Cs*severin decreased intracellular Ca^2+^ levels, resulting in the suppression of MPTP opening. Moreover, a drastic reduction in the mitochondrial translocation of Bax and release of Cyt c from mitochondria to the cytoplasm was observed. Furthermore, *Cs*severin inhibited the downstream caspase cascade, conferring protection from apoptosis.

Collectively, these data indicated that *Cs*severin can reduce calcium-mediated MPTP opening, which may be mediated through binding to actin and Ca^2+^. The inhibition of MPTP opening subsequently suppressed the translocation of Bax to mitochondria and the release of Cyt c from mitochondria, which in turn downregulates caspase 9 activities and caspase 3 protein expression, inducing obvious apoptotic suppression (Fig 7).

Taken together, these findings will be helpful to further illuminate the mechanism involved in tumorigenesis induced by *C*. *sinensis* infestation. Whether interventions according to this pathway are effective for the control of the disease progression is worthy of further exploration.
